# Engineered suppressor tRNAs enable precise translational control of genetic circuits in *E. coli*

**DOI:** 10.1093/nar/gkag623

**Published:** 2026-06-18

**Authors:** Xiaotong Wang, Jianping Xu, Yipeng Wang, Qingsheng Qi, Zhiguo Wang, Qian Wang

**Affiliations:** National Glycoengineering Research Center, Shandong University, Qingdao 266237, P. R. China; National Glycoengineering Research Center, Shandong University, Qingdao 266237, P. R. China; State Key Laboratory of Microbial Technology, Shandong University, Qingdao 266237, P. R. China; State Key Laboratory of Microbial Technology, Shandong University, Qingdao 266237, P. R. China; Zhejiang Key Laboratory of Medical Epigenetics, Institute of Aging Research, School of Basic Medical Sciences, Hangzhou Normal University, Hangzhou 311121, P. R. China; National Glycoengineering Research Center, Shandong University, Qingdao 266237, P. R. China

## Abstract

Precise and orthogonal regulation of genetic circuits is a central challenge in synthetic biology, particularly at the translational level where tools remain scarce. Here, we address this by engineering suppressor transfer RNAs (sup-tRNAs) charged with canonical amino acids to enable programmable nonsense mutation readthrough in *Escherichia coli*. Screening of 20 variants revealed a clear sup-tRNA design rule: readthrough efficiency is dictated by the similarity of the native tRNA anticodon to amber codon (CUA), as it preserves native aaRS interactions. We then demonstrate the power of this tool for advanced genetic circuit engineering. First, in a LacI-based biosensor, sup-tRNA regulation reduced background leakage by >77% and increased the induction dynamic range by 4.3-fold (from 6.67 to 28.68). Second, by dynamically balancing glycolytic flux through targeted *pykA* and *pykF* regulation, we increased the titer of N-acetylneuraminic acid by 66% (from 5.33 to 8.82 g/l) without compromising cell growth. Our work establishes engineered cAA-charged sup-tRNAs as a versatile, efficient, and cost-effective platform for precision translational control within genetic circuits, opening new avenues for biosensor optimization and metabolic engineering.

## Graphical Abstract

**Graphical Abstract:**
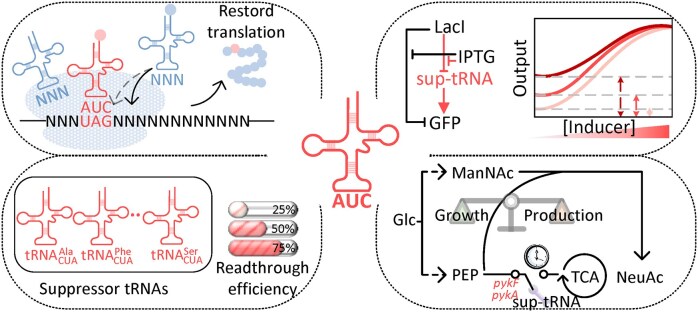

## Introduction

Synthetic biology aims to program cellular behaviors in a predictable manner through the design and construction of genetic elements, circuits, and metabolic pathways, thereby creating novel biological systems [1–3]. As synthetic biology applications continue to expand and diversify in scope, the successful execution of increasingly complex operations demands that individual genetic circuit components enable reliable and precise regulation of gene expression [4]. Such precise manipulation typically requires the regulation of intracellular protein abundance at multiple levels such as transcriptional (e.g. biosensors) [5], post-transcriptional (e.g. CRISPRa/i [6] and sRNA [7]) and post-translational level (targeted protein degradation) [8]. However, translational-level control, the direct regulation of protein synthesis, remains an underexplored layer in synthetic biology, creating a critical gap in the toolkit for fine-tuning genetic circuits, which is essential for applications like dynamic metabolic balancing and high-performance biosensors.

**Figure 1.**
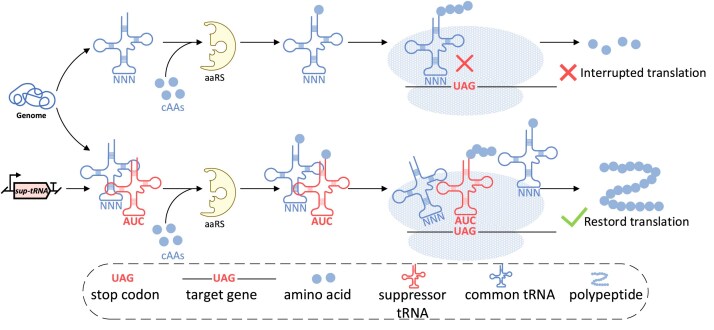
When encountering PTCs, sup-tRNA incorporate cognate amino acids into nascent peptide chains, restoring functional full-length proteins.

Suppressor transfer RNAs (sup-tRNAs) are defined as engineered tRNAs or natural variants that can read through premature termination codons (PTCs; Fig. 1), a foundational mechanism established in the 1980s [9–11] and further refined in subsequent studies [12]. Their therapeutic potential for genetic diseases and cancer is well recognized [9, 13–15]. In synthetic biology, a major application has been the engineering of orthogonal sup-tRNA/aminoacyl–tRNA synthetase pairs for incorporating noncanonical amino acids (ncAAs), enabling advanced functions such as genetic code expansion. Key methodological progress includes the automated design of orthogonal systems to coordinate multiple components and encode several ncAAs in one protein [16]. However, this orthogonal approach fundamentally depends on evolved, heterologous aminoacyl–tRNA synthetases, which can lead to efficiency and cost challenges that limit scalability in bacterial hosts like *Escherichia coli*. In contrast, suppression strategies utilizing endogenous, canonical amino acid-charged tRNAs offer a distinct, host-integrated alternative. This approach has been successfully applied in bacterial systems for metabolic regulation, highlighting its potential for efficient, large-scale applications within native cellular machinery. For instance, Ho *et al*. engineered a sup-tRNA-mediated feedforward loop to eliminate leaky gene expression in *E. coli*. Separately, Guo *et al*. developed a tRNA modification-based strategy to identify and enrich for amino acid overproducing strains [17, 18].

Yet, the design principles, efficiency determinants, and general utility of endogenous sup-tRNAs for precise translational control in synthetic circuits remain poorly understood and characterized, limiting their adoption as versatile regulators. To unlock this potential, we systematically engineered and characterized a library of canonical amino acid-charged sup-tRNAs in *E. coli*. We fine their key design rules and demonstrate their practical utility by solving two core challenges: enhancing biosensor performance (reducing leakiness, expanding dynamic range) and implementing dynamic metabolic control to boost product titer without impairing growth. This work establishes endogenous sup-tRNAs as a new class of precise, multifunctional components for translational regulation in synthetic biology.

## Materials and methods

### Bacterial strains and media

All bacterial strains employed in this study are cataloged in Supplementary Table S1. For cloning procedures, *E. coli* DH5α was cultivated in Luria–Bertani (LB) medium (10 g/l peptone, 5 g/l yeast extract, 10 g/l NaCl) at 37°C with shaking. System characterization studies utilized *E. coli* C321.ΔA.exp (The C321.ΔA.exp strain was purchase from Addgene), MG1655, BW25113, DH5α, BL21, and Nissle1917, cultured under identical conditions. Strain DN5 served as the production host for N-acetylneuraminic acid (NeuAc) fermentation. For fermentation studies, modified terrific broth (MTB) medium (12 g/l tryptone, 24 g/l yeast extract, 5 g/l NaCl) was employed. Antibiotics were supplemented as required: ampicillin (100 μg/ml), kanamycin (25 μg/ml), chloramphenicol (17 μg/ml), or spectinomycin (100 μg/ml).

### Plasmid construction

All plasmids used in this study are listed in Supplementary Table S2. Plasmid construction was performed in *E. coli* DH5α. The target gene fragment was amplified from the *E. coli* MG1655 genome. Polymerase chain reaction (PCR) amplification was performed using either Phanta Max Super-Fidelity DNA Polymerase or 2 × Taq Plus Master Mix II (Dye Plus) (Vazyme, Nanjing, China). DNA fragments and plasmids were purified using commercial kits from Omega Bio-tek. The purified products were subsequently assembled into the pACYC dute vector using the MultiF Seamless Assembly Mix (Cat# RK21020, ABclonal).

### Fluorescence measurement and data analysis

The sfGFP reporter fluorescence was quantified using a BioTek Synergy HTX multi-mode microplate reader. For kinetic analyses of growth and expression, overnight cultures were diluted to 2% (v/v) in 1.5 ml of LB medium in 24-well plates. OD_600_ and fluorescence (excitation: 485 nm; emission: 528 nm) were monitored simultaneously every 16 min for 18 h. All raw fluorescence readings were corrected by subtracting the autofluorescence of the plasmid-free *E. coli* C321.ΔA.exp host strain.

For endpoint measurements (used to characterize the LacI-based biosensor and to assay sup-tRNA readthrough efficiency), cultures were prepared similarly, induced with varying concentrations of isopropyl β-D-1-thiogalactopyranoside (IPTG), and incubated for 18 h before final OD_600_ and fluorescence readings were taken. These endpoint fluorescence values were processed identically, applying the same host-strain background subtraction.

The readthrough efficiency for each sup-tRNA was calculated from the corrected endpoint values as follows:


\begin{eqnarray*}
{\mathrm{ Efficiency}}\left( \% \right) = \frac{{{{F}_{{\mathrm{sfGFP(TAG)*\textit{suptRNA}}}}} - {{F}_{{\mathrm{C321}}{\mathrm{.}}\Delta {\mathrm{A}}.\exp }}}}{{{{F}_{\textit{sfGFP}}} - {{F}_{{\mathrm{C321}}{\mathrm{.}}\Delta {\mathrm{A}}.\exp }}}} \times 100\%
\end{eqnarray*}

This formula yields the percentage of full-length sfGFP produced by the sup-tRNA relative to the wild-type control, with host-derived background signal removed.

### Batch fermentation of NeuAc

A single colony on an agar plate was inoculated into 5 ml LB medium containing ampicillin and chloramphenicol and incubated overnight at 37°C. The culture was transferred to 50 ml LB medium containing ampicillin and chloramphenicol at a 2% (v/v) inoculum and cultured for 12 h. The culture was then inoculated into 50 ml fresh MTB medium containing ampicillin, chloramphenicol, and 20 g/l glucose at a 4% (v/v) inoculum in a 250 ml regular Erlenmeyer flask (without baffles), and cultivated to an OD_600_ of 0.4–0.6 at 30°C and 220 rpm. Then 200 µM IPTG was added for induction. Samples were taken every 12 h for analysis, and 30% NH_3_·H_2_O was added to maintain the pH at ∼7.0. The fermentation was terminated after 96 h.

### Analytical methods

OD_600_ was measured using a spectrophotometer (Shimazu, Japan). Fermentation samples were centrifuged at 12 000 rpm for 4 min, and the supernatants were used for extracellular metabolite analysis. Glucose was measured using a Biosensors Analyzer (Jinan, China). NeuAc, N-acetylglucosamine (GlcNAc), and N-acetylmannosamine (ManNAc) were quantitatively determined by high-performance liquid chromatography (Shimazu, Japan) equipped with a refractive index detector (RID-10 A) (Shimadzu, Japan) and an Aminex HPX-87H ion exclusion column (Bio-Rad, USA), as described previously. The mobile phase is 5 mM sulfuric acid and the flow rate is 1 ml/min.

### Insert TAG on the genome

Genome editing was performed through the following steps: (i) Selection of 90-bp insertion site-containing fragments from the *E. coli* genome, followed by TAG codon insertion and long primer synthesis; (ii) Sequential electroporation of pEcCas plasmid into target strains, then co-delivery of long primers and pEcgRNA; (iii) Selection on kanamycin/spectinomycin plates after culture recovery; (iv) Verification of edited clones by colony PCR; (v) Final curing of pEcCas and pEcgRNA plasmids.

### Molecular simulation

The binding structures of AARS and corresponding tRNA were built with AlphaFold 3 [19]. The models which outperform in the pLDDT (predicted local distance difference test), ipTM (the interface/predicted TM-score), and pTM (predicted template modeling score) were chosen as input structures for molecular dynamics (MD) simulations. All MD calculations were conducted by employing the AMBER 24 software [20]. The FF19SB and OL3 force fields were applied for AARS and tRNA, respectively. The binding complexes were individually immersed into the center of a truncated octahedron box of Optimal Point Charge (OPC) water molecules with a margin distance of 10.0 Å, Na+ counterions were added to keep systems in electric neutrality. Following the calculation protocol and parameters reported in previous study [21], each system was simulated with coordinate snapshots saved every 10 ps to record MD trajectory. The binding affinity between AARS and tRNA was evaluated through the molecular mechanics/generalized Born surface area (MM/GBSA) calculation [22]. A total of 200 snapshots were evenly extracted from the last 10 ns of MD trajectory for the MM/GBSA calculations. The calculated binding free energies (ΔG_bind_) comprised contributions from van der Waals energy (E_vdW_), electrostatic energy (E_ele_), solvation free energy including a polar part (G_GB_) and a nonpolar part (G_SA_), and the entropy (TΔS). Though the entropy item can be approximately estimated through normal mode analysis [22], such treatment rarely leads to improvement in the correlation with experiments [23]. Therefore, the entropy contribution was not included in this study. The change in binding affinity due to the mutation was defined as ΔΔG_bind_ = ΔG_bind_(tRNA-original) − ΔG_bind_(tRNA-modified), where positive values indicate enhanced binding of the sup-tRNA (Supplementary Tables S5 and S6).

### Statistical analysis

All data are presented as mean values ± standard deviation (SD) from three independent biological replicates (*n* = 3). Statistical significance was determined using one–way Analysis of Variance (ANOVA) followed by Tukey’s multiple–comparisons test (GraphPad Prism 9.0). A *P*–value <.05 was considered statistically significant (*P <.05, **P <.01, ***P <.001, ****P <.0001).

## Result

### Characterization of engineered sup-tRNAs with high readthrough efficiency

To develop sup-tRNAs with strong termination codon readthrough efficiency, we engineered the natural tRNAs of 20 amino acids in *Escherichia coli*. First, based on the codon preference of *E. coli*, tRNAs corresponding to the optimal codons of the 20 canonical amino acids (cAAs) were selected as candidates (their identities and genomic details are provided in Supplementary Table S4, and the codon correspondence is listed in Supplementary Table S3), For amino acids with multiple isodecoders (Leu, Thr, Tyr, Val), we chose to engineer all isodecoder tRNAs simultaneously into sup-tRNAs to systematically evaluate their readthrough efficiency. Meanwhile, to minimize the impact of sup-tRNA-mediated suppression of translation termination on other genes, we chose the rarest termination codon (TAG, Amber) in *E. coli* as the termination codon to be recognized [24].

**Figure 2.**
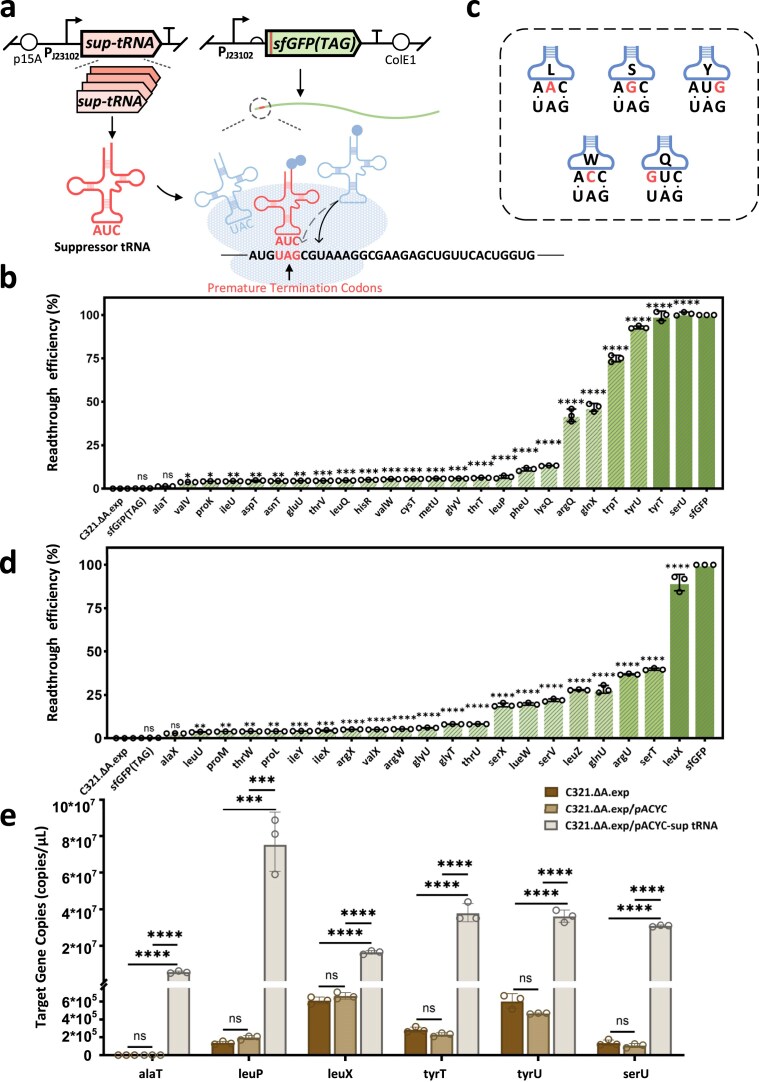
Characterization of 20 engineered sup-tRNAs and quantification of UAG readthrough efficiency. (**a**) Schematic of the experimental design: each sup-tRNA was co-expressed with the sfGFP(TAG) reporter, enabling quantification of readthrough efficiency via restoration of GFP fluorescence. (**b**) Quantified readthrough efficiency (mean ± SD, *n* = 3 biological replicates), represented as the recovery of sfGFP fluorescence in the C321.ΔA.exp. All fluorescence values were background-corrected by subtracting the autofluorescence of the empty host strain (C321.ΔA.exp), as detailed in the ‘Materials and methods’ section. Statistical significance was determined using one-way ANOVA with Tukey’s multiple comparisons test, based on the corrected data. *P <.05, **P <.01, ***P <.001, ****P <.0001 compared to the control. (**c**) Anticodon sequences of 5 near-cognate tRNAs that partially base-pair with UAG, with mismatched bases highlighted in red. (**d**) Quantified recovery of sfGFP fluorescence (mean ± SD, *n* = 3 biological replicates) mediated by another twenty-two engineered tRNAs. (**e**) Absolute intracellular abundance of six representative sup-tRNAs in *E. coli*, as determined by quantitative Reverse Transcription Quantitative PCR (RT–qPCR) (mean ± SD, *n* = 3). Statistical significance was determined using one-way ANOVA with Tukey’s multiple comparisons test, based on the corrected data. *P <.05, **P <.01, ***P <.001, ****P <.0001 compared to the control.

By modifying the anticodon loops of candidate tRNAs to CUA to pair with the termination codon UAG, we engineered 20 sup-tRNAs capable of UAG readthrough. We selected sfGFP as the reporter gene and inserted a TAG after the sfGFP start codon to construct sfGFP(TAG). This causes immediate translational termination and minimizes the accumulation of abbreviated translation products (since sfGFP is highly robust, insertions or fusions at its N-terminus do not affect its overall function) [25]. Then co-expression of each sup-tRNA with sfGFP(TAG) enabled quantification of readthrough efficiency via fluorescence recovery, the fluorescence intensity of sfGFP(TAG) is positively correlated with the readthrough efficiency of sup-tRNAs. The plasmids harboring 20 types of sup-tRNAs and a plasmid carrying sfGFP(TAG) were transformed into strain *E. coli* C321.ΔA.exp [26], respectively. C321.ΔA.exp is a recoded *E. coli* strain with innate amber termination abolished through the removal of amber codons from the genome and the deletion of release factor 1. The recovery efficiency of sup-tRNAs on translation was verified by detecting the fluorescence recovery of sfGFP (TAG) (Fig. 2A and B).

Compared to the wild-type sfGFP control (set at 100%), more than half of the engineered sup-tRNAs exhibited low termination readthrough efficiency (<10%). Moderate efficiency (10%–45%) was observed for the *pheU, lysQ, leuP*, and *argQ* variants. Four sup-tRNA variants exhibited a readthrough efficiency exceeding 45%: *glnX* (46.8%), *trpT* (74.9%), *tyrU* (92.8%), *tyrT* (99.4%), and *serU* (100%). A consistent rule emerged from our initial screen: all high-efficiency sup-tRNA variants (>45% readthrough) derive from tRNAs whose native anticodons differ from CUA by just one nucleotide. This trend, encompassing the tRNAs for four amino acids Ser, Tyr, Trp, and Gln, directly led to the hypothesis that minimal anticodon alteration preserves native aaRS interactions to achieve high function (Fig. 2C). To validate this hypothesis, we engineered all remaining tRNAs in *E. coli* into sup-tRNAs and tested them using the sfGFP(TAG) reporter (Fig. 2D). The results were consistent with our expectations: only the sup-tRNA derived from leuX, which shares similar characteristics, exhibited high readthrough efficiency. The resulting sup-tRNA confirmed the hypothesis, achieving a high readthrough efficiency of 89.7%, solidifying the evidence that minimal anticodon alteration strongly supports high readthrough performance. For tRNAs with two or three nucleotide mismatches, the majority exhibited readthrough efficiencies below 5%, while approximately one-quarter fell within the 5%–45% range. Thus, while a higher mismatch number generally correlates with lower readthrough efficiency, the relationship is not strictly linear, suggesting that additional factors also influence readthrough performance (Supplementary Fig. S1).

To investigate the determinants of readthrough efficiency among different sup-tRNAs, we selected 1–2 sup-tRNAs derived from the tRNAs of each amino acid for molecular dynamics simulations, and first examined how the CUA anticodon substitution affected aaRS binding affinity (ΔΔG_bind_) through molecular dynamics simulations (Supplementary Fig. S2).Most high-efficiency sup-tRNAs (corresponding to Ser, Tyr, Trp, Gln, and Leu) displayed positive ΔΔG_bind_ values, indicating enhanced binding affinity. Exceptions were noted: *argQ* maintained moderate-to-high readthrough despite negative ΔΔG_bind_, while the asnT-aaRS pair showed increased affinity without high efficiency (Supplementary Fig. S3A and B).

To assess other contributing factors, we quantified the intracellular abundance of six representative sup-tRNAs (*alaT, leuP, leuX, tyrT, tyrU*, and *serU*) using absolute RT–qPCR, with a standard curve constructed for accurate quantification (Supplementary Fig. S4). A general positive correlation was observed between expression level and readthrough efficiency, with *leuP* being a notable exception, exhibiting the highest abundance but only moderate activity (Fig. 2E). And the expression levels ofthree sup-tRNAs (*tyrT/tyrU/serU*) were further validated using northern blot (Supplementary Fig. S5), which basically supported the RT-qPCR results, showing a positive correlation between band intensity (abundance) and readthrough efficiency.

Collectively, these results indicate that readthrough efficiency is governed by multiple factors: anticodon compatibility with CUA, the resulting change in aaRS binding affinity, and intracellular expression levels. Based on this integrated framework, the top-performing variants—*supD* (derived from *serU*), *supT*, and *supC* (derived from *tyrT/U*)-were selected for all subsequent applications.

### Engineered sup-tRNAs enhance LacI biosensor performance across model *E. coli* hosts

As an emerging synthetic biology tool, the role of biosensors in increasingly complex biological functions is important. The dose-response curve is a key method for evaluating the performance of biosensors and shows the parameters that affect their application, such as dynamic range, background leakage, response threshold and sensitivity. In particular, background leakage represents the expression of the reporter gene in the uninduced state [27, 28], and affects the sensitivity and accuracy of detection. Scientists have therefore been committed to reducing background expression in biosensors.

Based on our initial screening of high-performance sup-tRNAs (*supD, supT*, and *supC*), we engineered a translational-level regulation system and applied these three engineered sup-tRNAs to optimize biosensor performance. We selected the widely used LacI-based biosensor, which employs the small-molecule inducer IPTG (Supplementary Fig. S6) to regulate gene expression in biosynthetic pathways. Within this system, the biosensor promoter *P_lac-O_* contains the *lac* promoter and a *lacO* operator downstream of −10 region. The sup-tRNAs were placed under the control of the biosensor promoter *P_lac-O_*, while a PTC (TAG) was introduced into the reporter gene *sfgfp. P_lac-O_* controls expression of both of sfGFP(TAG) and sup-tRNA. In the absence of IPTG, the TAG codon halts translation of leaky messenger RNA (mRNA) transcripts, and the basal expression level of sup-tRNA is insufficient to support full-length sfGFP(TAG) translation. This design significantly reduces background leakage. Upon IPTG induction, both sfGFP(TAG) and sup-tRNA expression increases, enabling the sup-tRNA to suppress the TAG stop codon and restore full-length sfGFP(TAG) translation to its original level. Consequently, this approach reduces biosensor background leakage without compromising maximum activation, thereby improving the dynamic range.

**Figure 3.**
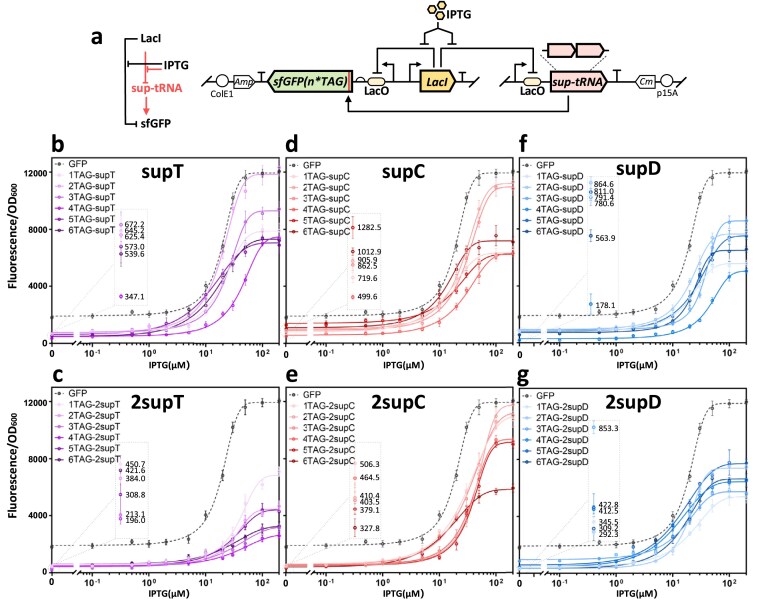
Optimization of a LacI-based biosensor by modulating sup-tRNA variants and TAG-codon numbers. (**a**) Schematic of the optimization strategy, involving the modulation of gene copy numbers of sup-tRNA variants and the number of TAG codons in the sfGFP reporter. (**b–g**) Assessment of biosensor performance mediated by different sup-tRNAs. Shown are the dose-response curves for systems with single- or dual-copy expression of (**b, c**) *supT* (*tyrT*-derived), (**d, e**) *supC* (*tyrU*-derived), and (**f, g**) *supD* (*serU*-derived), each paired with sfGFP reporters containing varying numbers of TAG codons (*n* = 6). (Inset: background leakage of each system under uninduced conditions.)

The results showed that the unmodified LacI-based biosensor exhibited a 6.67-fold induction across 0–200 μM IPTG. In theory, the efficiency of translational termination at the TAG codon depends on its frequency, Increasing the number of TAG codons would reduce background leakage but could potentially impair maximum activation. Conversely, increasing the expression level of sup-tRNA would help preserve maximum activation but might also increase background expression. In order to identify the optimal balance, we systematically characterized the combinations of 1–6 TAG codons in sfGFP(TAG) and 1–2 copy numbers of each sup-tRNA in *E. coli* C321.ΔA.exp (Fig. 3A).

According to the type and number of sup-tRNAs in the combinations, as well as the number of TAGs in sfGFP(TAG), we named the combinations as n*sup-tRNA-n*TAG respectively. The results showed that the maximum outputs of supT-2TAG, supC-2TAG, supC-3TAG, 2supC-1TAG, 2supC-2TAG, and 2supC-3TAG were almost equal to the maximum output of wild-type sfGFP. The leaky expressions of supT-4TAG, 2supT-n*TAG, supC-4TAG, 2supC-n*TAG, supD-4TAG, 2supD-1TAG, 2supD-2TAG, 2supD-4TAG, 2supD-5TAG, and 2supD-6TAG were all significantly reduced to below 30% of that of Lac-GFP control (Fig. 3B–G). Among them, 2supC-2TAG showed the best performance. It exhibited minimal leaky expression (410.4 a.u.) without inducer, while maintaining maximum output equivalent to the original biosensor at 200 μM IPTG. This yielded a significantly enhanced maximum induction fold of 28.68 (Fig. 3E). These results establish that the 2supC-2TAG configuration provides the most effective optimization for reducing leakage and preserving maximum output in the LacI-based biosensor within *E. coli* C321.ΔA.exp.

**Figure 4.**
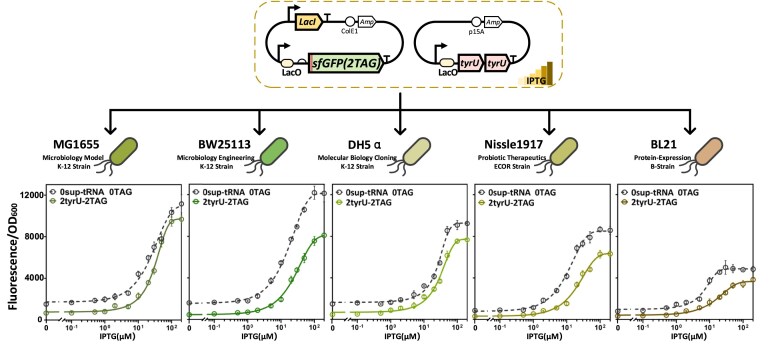
The LacI-based biosensor modified with the optimal 2supC-2TAG combination was introduced into five common laboratory strains (MG1655, BW25113, DH5α, Nissle1917, and BL21).

While the above results demonstrate that sup-tRNAs can effectively optimize the performance of biosensors, the recoded *E. coli* C321.ΔA.exp represents a specialized chassis. To assess the universality of our findings in standard model *E. coli* hosts, we introduced the LacI-based biosensor modified with the optimal 2supC-2TAG combination into five common laboratory strains: MG1655, BW25113, DH5α, Nissle1917, and BL21. Characterization revealed that in these strains, the basal leakage expression of the optimized biosensor (in the absence of IPTG) was reduced to 44.2%, 28.2%, 31.3%, 35.7%, and 41.9%, respectively, relative to the leakage level of the original, unmodified biosensor in each corresponding strain (set as 100%). This confirms a substantial reduction in background signal across all hosts. Correspondingly, the induction fold increased to 14.40-fold, 17.78-fold, 16.44-fold, 20.62-fold, and 11.60-fold (Fig. 4). These fold-change values were calculated from background-subtracted luminescence readings. Importantly, this optimization did not cause any detectable growth defects in these strains (Supplementary Fig. S7). These findings indicate that the biosensor optimized with engineered sup-tRNA exhibits significantly improved performance across diverse model *E. coli* strains. This demonstrates that the ability of engineered sup-tRNA to regulate translation remains robust across diverse genetic backgrounds, exhibiting broad functional portability.

### Growth-phase-dependent sup-tRNA control optimizes PEP partitioning for enhanced NeuAc Biosynthesis

**Figure 5.**
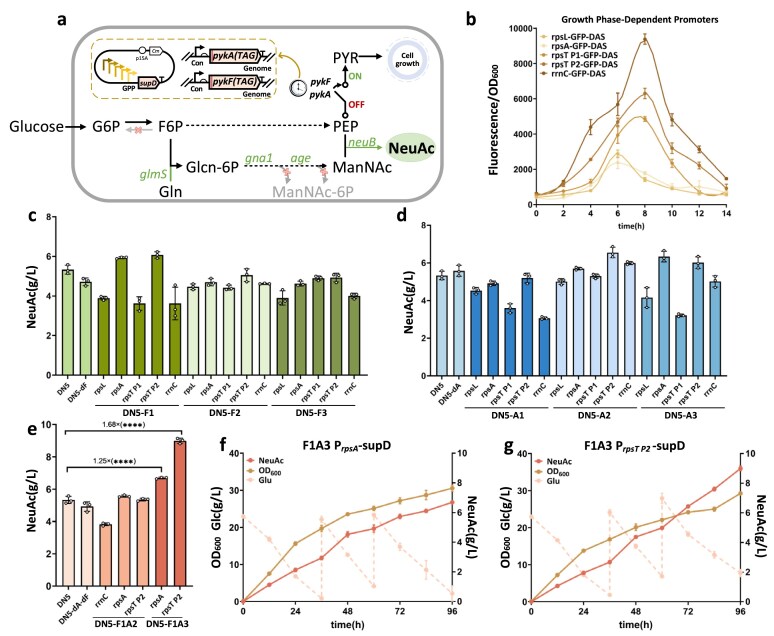
Growth-phase-dependent sup-tRNA control optimizes PEP partitioning for enhanced NeuAc biosynthesis. (**a**) The biosynthetic pathway of NeuAc in *E. coli* is depicted with gene knockouts marked in red and gene overexpressions in green. GPP-supD dynamically modulates metabolic flux distribution during the fermentation process. (**b**) Characterization of five GPP variants with varying expression intensities. (**c**) Fermentation performance of *pykF*-targeted engineered strains. (**d**) Fermentation performance of *pykA*-targeted engineered strains. (**e**) Fermentation profiling of *pykF/pykA* double-gene engineered strains. (**f**). fermentation performance of the F1A3 P*_rpsA_*-*supD* genetic module. (**g**) Fermentation performance of the F1A3 P*_rpsT_-supD* genetic module.

The preceding experiments have established the capacity of engineered sup-tRNAs to precise regulate gene expression and successfully optimize biosensor performance. To explore the broader applicability of this toolkit, we investigated its potential within the biosynthesis of NeuAc. NeuAc is a functionally significant sialic acid widely present in glycoproteins and glycolipids on animal cell membranes [29]. It has demonstrated roles in promoting infant brain and bone development and enhancing memory and learning capabilities [30]. In *E. coli*, the dominant NeuAc biosynthesis occurs via the acylglucosamine pathway, proceeding through: glucose→GlcNAc-6P→ManNAc, ManNAc is converted to NeuAc via a NeuB-mediated condensation reaction with phosphoenolpyruvate (PEP) (Fig. 5A). Simultaneously, PEP represents a critical intracellular metabolic node. It is primarily catalyzed by pyruvate kinase to generate pyruvate, which feeds directly into the tricarboxylic acid cycle for generating ATP and reducing power to support cellular growth. Consequently, competition for PEP between central metabolism (supporting growth) and the NeuAc biosynthetic pathway dictates its allocation and constitutes a fundamental bottleneck limiting efficient NeuAc production.

A previously constructed NeuAc-producing strain *E. coli* DN5 [31] (DH5α *Δnag ΔackA ΔpoxB ΔldhA Δnan*) harboring the plasmid pBac43 (containing genes encoding NeuAc synthase, GlcNAc 2-epimerase, GlcN-6-P N-acetyltransferase and feedback-resistant GlcN-6-P synthase) was able to accumulate 5.33 g/l NeuAc in shake flask. This strain also accumulated more precursors GlcNAc and ManNAc, probably due to the relatively insufficient supply of precursor PEP. Direct knock out of the pyruvate kinase genes *pykF* and *pykA* caused cell growth defects, which instead reduced the production (Supplementary Fig. S8).

To resolve this metabolic bottleneck, we engineered a growth-responsive translational control system utilizing growth phase-dependent promoters (GPPs) [32] to promote the transcription of engineered sup-tRNA. These GPPs activate sup-tRNA transcription during the growth phase and downregulate it during stationary phase, enabling dynamic regulation of target genes *pykF* and *pykA* within the genome of the strain DN5. Then this strategy was precisely implemented by selecting five varying strengths’ GPPs—rpsL, rpsA, rpsT P1, rpsT P2, rrnC (Fig. 5B) to drive *supD* expression while introducing premature TAG stop codons near the start codons of *pykF* and *pykA*. Specifically, we replaced the first to third codons immediately following the start codon of each gene with the amber stop codon (TAG), generating strains DN5-F1, DN5-F2, DN5-F3 (for *pykF* modifications) and DN5-A1, DN5-A2, DN5-A3 (for *pykA* modifications) (Supplementary Table S2). As controls, we constructed strains with complete deletions of *pykF* (DN5-dF) and *pykA* (DN5-dA). This design establishes a metabolic switch: during growth, *supD* expression permits readthrough of the TAG codons, enabling pyruvate kinase expression to direct PEP flux toward cell growth; during stationary phase, *supD* depletion causes translation termination at TAG, blocking pyruvate kinase production and diverting PEP flux toward NeuAc biosynthesis (Fig. 5A).

Fermentation analysis revealed distinct outcomes. DN5-dF reduced NeuAc titer to 4.71 g/l. In contrast, DN5-F1 exhibited increased titers of 5.93 g/l and 6.07 g/l when regulated by the P*_rpsA_*-*supD* and P*_rpsT-P2_*-*supD* systems, respectively (Fig. 5C). Similarly, DN5-dA yielded 5.58 g/l NeuAc, while strains with *pykA* TAG modifications showed further improvement: DN5-A2 produced 5.70 g/l, 6.55 g/l, and 5.99 g/l under P*_rpsA_*-*supD*, P*_rpsT-P2_*-*supD*, and P*_rrnC_*-*supD* regulation, respectively, and DN5-A3 yielded 6.34 g/l and 6.01 g/l under P*_rpsA_*-*supD* and P*_rpsT-P2_*-*supD* regulation (Fig. 5D). This result indicated that engineered sup-tRNA regulated translation inhibition of PykA (PykII) surpasses PykF (PykI) in enhancing NeuAc production, which may due to that PykI downregulation compromises growth via essential metabolic roles [33], while PykII offers regulatory flexibility for growth-uncoupled flux optimization.

To further enhance NeuAc production, we integrated the above modification strategies by constructing combinatorial strains DN5-F1A2 and DN5-F1A3. In these engineered strains, the *pykF* gene was modified to insert a single TAG codon after ATG. Concomitantly, the *pykA* gene was engineered to contain either two (strain DN5-F1A2) or three (strain DN5-F1A3) TAG codons after ATG. Then we employed the regulatory systems P*_rpsA_*-*supD*, P*_rpsT-P2_*-*supD*, and P*_rrnC_*-*supD* to control gene expression levels during shake flask fermentation (Fig. 5E). Fermentation analysis revealed that strain DN5-F1A3, under P*_rpsA_*-*supD* regulation, achieved a NeuAc titer of 6.72 g/l at 96 h (Fig. 5F), corresponding to an overall NeuAc yield of 0.101 g/g glucose and a specific yield of 8.29 mg/g glucose/g Cell Dry Weight (CDW). When regulated by P*_rpsT-P2_*-*supD*, the same strain DN5-F1A3 produced 8.82 g/l NeuAc (Fig. 5G), with an overall yield of 0.141 g/g glucose and a specific yield of 12.1 mg/g glucose/g CDW. Compared to the parental strain DN5, the P*_rpsA_*-*supD* regulated strain showed an 19% improvement in overall yield, while the P*_rpsT-P2_*-*supD* regulated strain achieved a 66% improvement (Supplementary Table. S7). During fermentation, the abundance of *supD* regulated by the GPPs GPP (rpsA and rpsT P2) exhibited a distinct rise–and–fall pattern along with cell growth (Supplementary Fig. S9). These enhancements occurred without compromising cellular growth rate (Supplementary Fig. S10). These results demonstrate that the sup-tRNA-mediated genetic circuit can effectively regulate gene expression and dynamically balance the cellular resources between heterologous protein production and host cell growth. This approach establishes a versatile strategy applicable to the synthesis of diverse high-value chemicals.

## Discussion

Precise control of translational elongation offers a new dimension for gene expression regulation, holding promise to address challenges such as leakage control and dynamic balancing that are difficult to tackle with traditional transcriptional regulation [34–36]. This study reports a translation-regulating tool based on the engineering of endogenous tRNAs. By systematically evaluating the termination codon readthrough performance of sup-tRNAs corresponding to all tRNAs in *E. coli* and successfully applying them to biosensor optimization and metabolic pathway remodeling, we have confirmed the feasibility of achieving precise control at the translation level.

Screening of sup-tRNAs revealed that their readthrough efficiency is closely correlated with the sequence similarity between the anticodon of the source tRNA and CUA: all high-efficiency variants (readthrough >45%) originated from tRNAs whose native anticodon differs from CUA by only one nucleotide. This finding aligns with prior knowledge that minimal modifications in the anticodon region help maintain basic recognition between the tRNA and its cognate aminoacyl–tRNA synthetase (aaRS). Furthermore, we also found that the sequence of the template tRNA itself affects the readthrough efficiency of the engineered sup-tRNA, as evidenced by the fact that sup-tRNAs derived from different isoacceptors of the same amino acid also exhibit differences in readthrough efficiency. This is likely because a higher degree of overall structural integrity of the template tRNA enables the modified sup-tRNA to better retain its functionality. Subsequent binding free energy calculations further indicated that for most variants following this pattern (e.g. Ser, Tyr, Trp), the CUA substitution enhanced binding affinity to their aaRS (ΔΔG_bind_ > 0), providing a partial energetic basis for high-efficiency readthrough. However, this correlation is not absolute. For instance, *argQ* exhibited relatively high efficiency despite showing no advantage in binding affinity, suggesting the existence of other compensatory mechanisms. Conversely, enhanced affinity alone is not sufficient for high readthrough efficiency. These findings demonstrate that while binding affinity is an important factor, sup-tRNA function is also governed by additional regulatory mechanisms.

The study also examined the role of intracellular expression levels. Although tRNA abundance generally positively correlates with readthrough efficiency, the disparity between cases such as *leuP* (high abundance, moderate efficiency) and *serU* (moderate abundance, highest efficiency) indicates that the intrinsic compatibility of the tRNA with the translational machinery is more critical than expression level alone. Therefore, the design of high-performance sup-tRNAs requires an integrated consideration of three aspects: (i) anticodon compatibility with CUA (maintaining basic recognition), (ii) the resulting change in aaRS binding affinity (providing energetic drive), and (iii) the achievable intracellular expression level (determining output strength). This multi-factor framework provides a unified explanation for the main findings and exceptions observed in this study.

Guided by this understanding, we successfully applied high-efficiency sup-tRNAs to address two practical problems. In biosensors, leak suppression at the translational level avoids the inherent basal noise of transcriptional regulation, offering a new strategy for constructing high signal-to-noise ratio systems. In metabolic engineering, dynamically balancing flux distribution through translational regulation enhanced target product synthesis without compromising cell growth. Furthermore, this study observed differential regulatory effects of sup-tRNAs on highly homologous isozymes (e.g. PykA and PykF), highlighting the unique potential of translational control tools for fine-tuning metabolic networks.

This strategy offers inherent advantages such as a direct mechanism, good host compatibility, and minimal genetic perturbation. However, it is also subject to limitations including the influence of mRNA sequence context and potential competition with endogenous release factors. Consequently, it is particularly suitable for synthetic biology scenarios requiring high precision, dynamic adjustability, or strict constraints on genetic modification depth.

In summary, by establishing and applying a rational design-validation framework for sup-tRNAs, this study develops translational elongation into a programmable regulatory dimension. Compared to systems relying on ncAAs, this strategy is more readily generalizable. Compared to traditional transcriptional regulation, it demonstrates unique value in addressing leakage and achieving dynamic balance, providing new tools and insights for building efficient and controllable cell factories.

## Supplementary Material

gkag623_Supplemental_File

## Data Availability

All data generated in the present study and included in this article and its supplementary information files are available from the corresponding author upon reasonable request.
