# Supplementary material for: Engineered suppressor tRNAs enable precise translational control of genetic circuits in E. coli
Source: Nucleic Acids Res. 2026 Jun 18;54(11):gkag623. doi: 10.1093/nar/gkag623 (PMC13276491; doi:10.1093/nar/gkag623)
Supplement: gkag623_Supplemental_File [file gkag623_supplemental_file.pdf]

Supplementary Information

Engineered suppressor tRNAs enable precise translational control of genetic circuits in *E. coli*

Xiaotong Wang[1], Jianping Xu[1], Yipeng Wang[2], Qingsheng Qi[2], Zhiguo Wang[3]*, Qian Wang[1]*

[1]National Glycoengineering Research Center, [2]State Key Laboratory of Microbial Technology, Shandong University, Qingdao 266237, P. R. China

[3]Zhejiang Key Laboratory of Medical Epigenetics, Institute of Aging Research, School of Basic Medical Sciences, Hangzhou Normal University, Hangzhou, 311121, P. R. China

*Corresponding author:
Qian Wang. Tel: +86532-58631580, Email: qiqi20011983@gmail.com;
Zhiguo Wang. Email: zhgwang@hznu.edu.cn.

Supplementary Figure S1

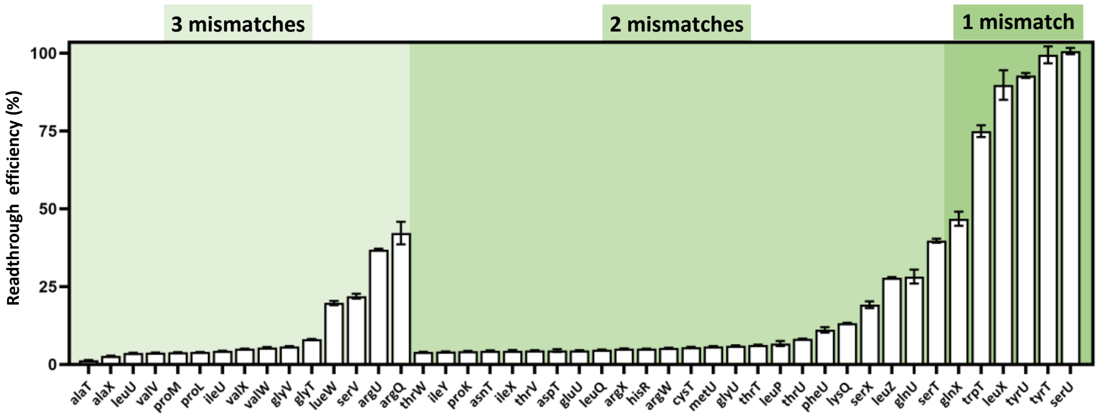

Ranking of sup-tRNA readthrough efficiencies grouped by the number of mismatches (1-3) between the native anticodon and the TAG stop codon. The engineered sup-tRNAs are categorized into three groups based on the number of nucleotide differences (1, 2, or 3) between their native anticodon and the TAG codon, and within each group, the variants are ordered by increasing readthrough efficiency.

Supplementary Figure S2

Molecular docking models of all aaRS-tRNA complexes

tRNA	aaRS Class	tRNA-aaRS-original	tRNA-aaRS-modified
leuX	Class I	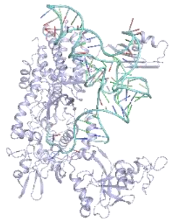	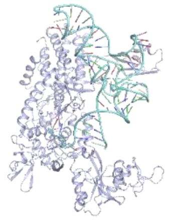
leuP	Class I	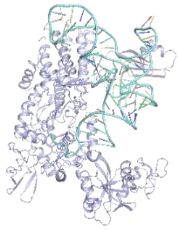	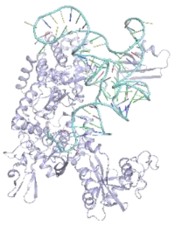
argQ	Class I	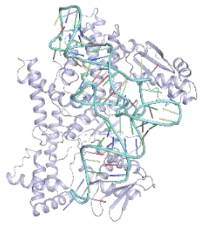	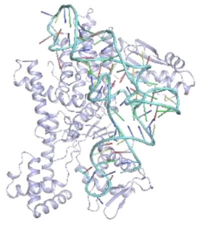
cysT	Class I	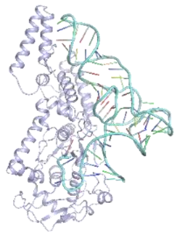	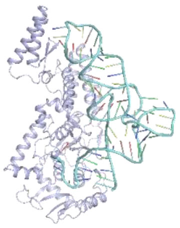
glnX	Class I	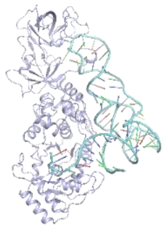	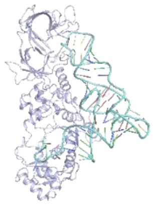
gluU	Class I	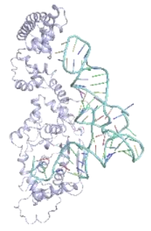	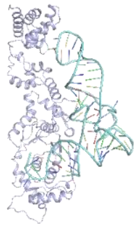

ileX	Class I	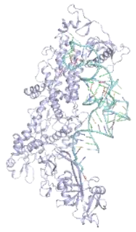	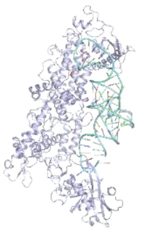
metU	Class I	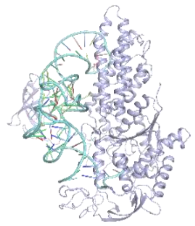	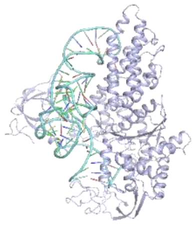
trpT	Class I	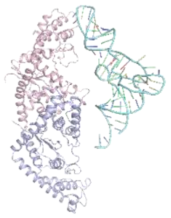	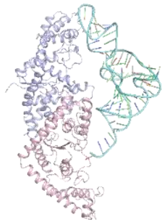
tyrT	Class I	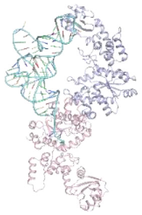	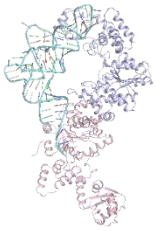
tyrU	Class I	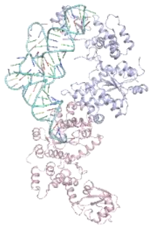	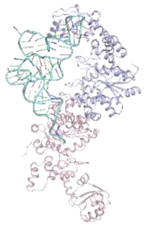
valW	Class I	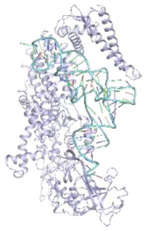	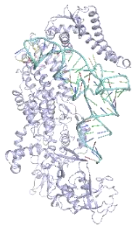
serU	Class II	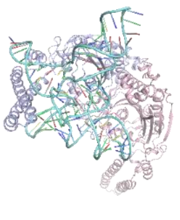	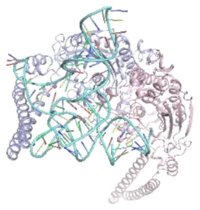

alaT	Class II	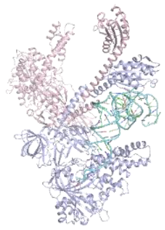	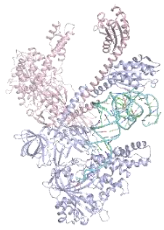
asnT	Class II	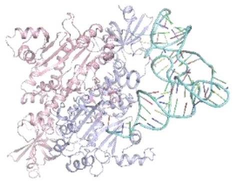	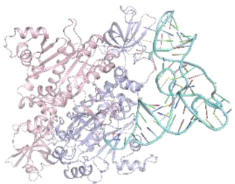
aspT	Class II	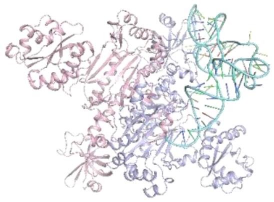	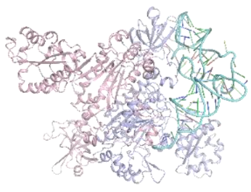
proK	Class II	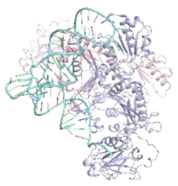	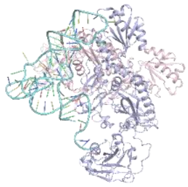
thrT	Class II	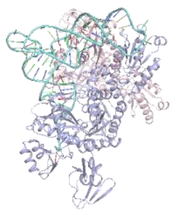	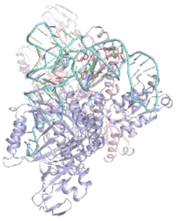
glyV	Class II	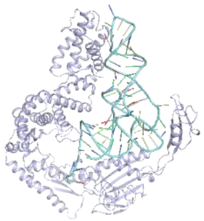	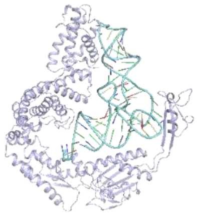
hisR	Class II	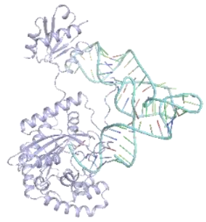	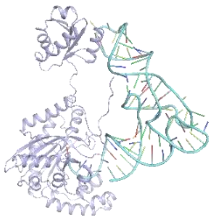

lysQ	Class II	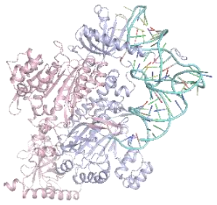	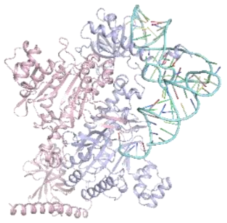
pheU	Class II	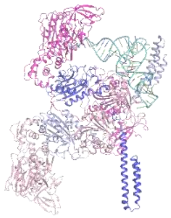	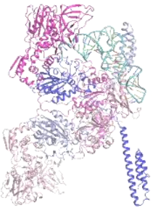

Conformations of the equilibrated binding structures of AARS–tRNA derived from MD simulations. The bases of U, A, G, and C in tRNA are shown as yellow, red, green, and blue sticks, respectively.

Supplementary Figure S3

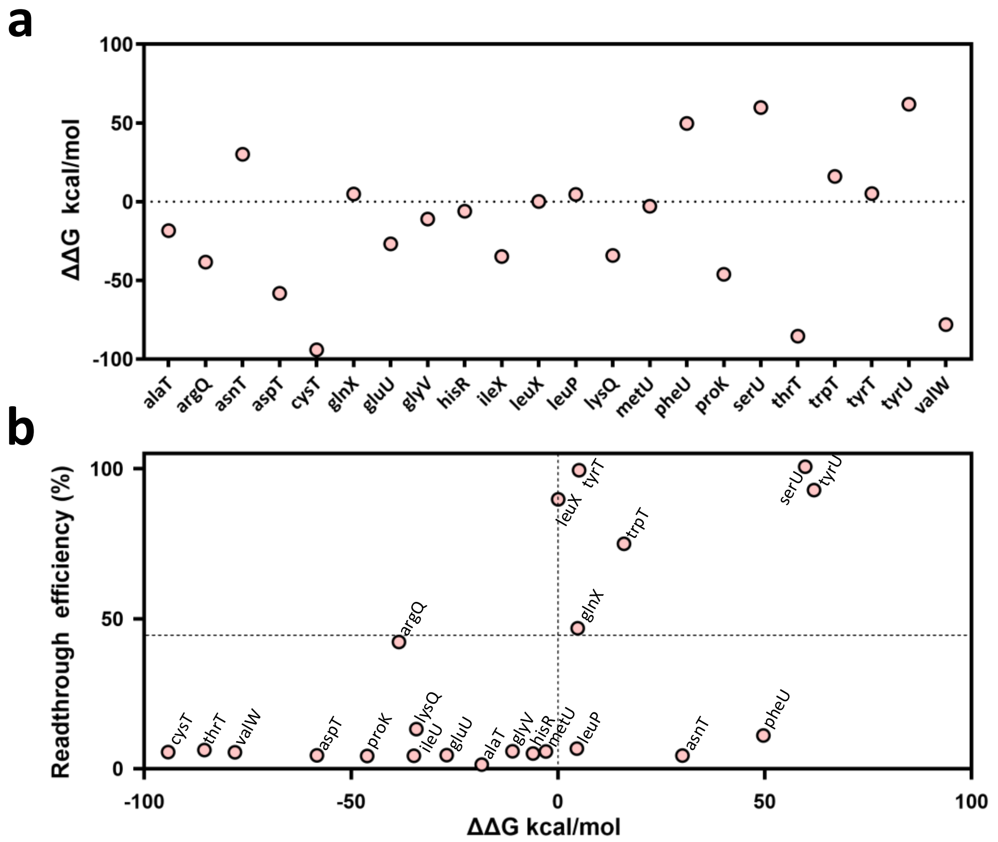

Supplementary Fig 3. Correlation between $\Delta\Delta G_{bind}$ and readthrough efficiency of sup-tRNA variants. a: The change in binding affinity due to the mutation was defined as $\Delta\Delta G_{bind} = \Delta G_{bind}$(tRNA-original) - ΔG_{bind}(tRNA-modified), where positive values indicate enhanced binding of the sup-tRNA. b: A scatter plot illustrating the relationship between readthrough efficiency and the change in binding affinity ($\Delta\Delta G_{bind}$) for each sup-tRNA variant. The x-axis represents $\Delta\Delta G_{bind}$ (kcal/mol), and the y-axis represents readthrough efficiency (%). The six sup-tRNAs within the dashed box exhibit higher readthrough efficiency, sup-tRNAs with higher readthrough efficiency show more positive $\Delta\Delta G_{bind}$ values.

Supplementary Figure S4

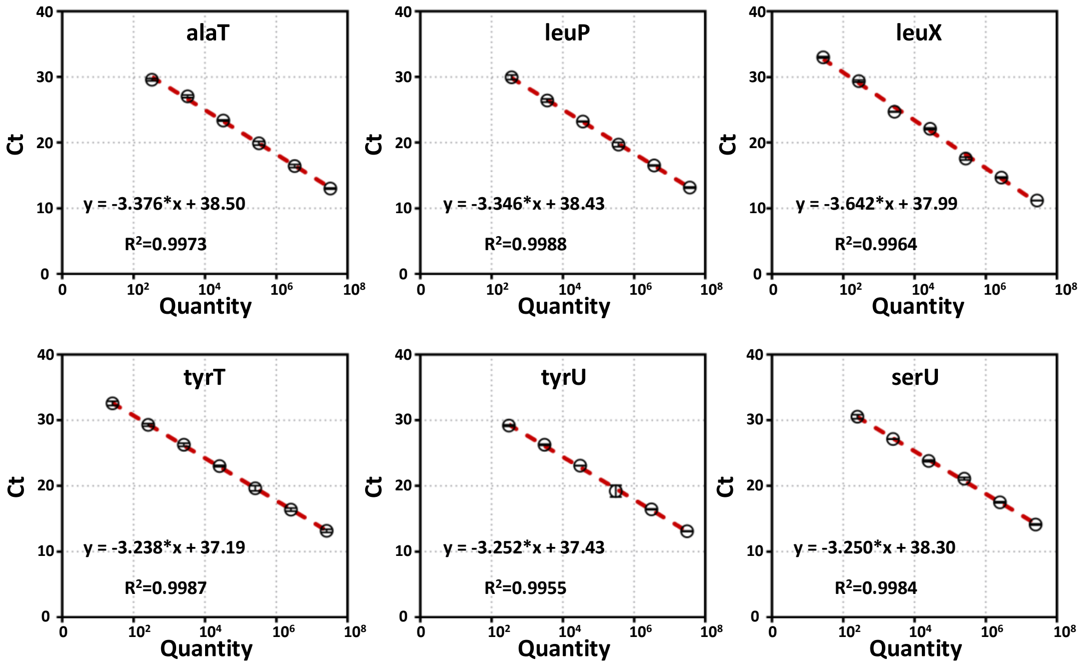

Standard curves used for the absolute quantification of the indicated sup-tRNAs. First, a plasmid containing the target sequence but not expressing sup-tRNA is constructed to serve as the standard template. The plasmid is accurately quantified and subjected to a 10-fold serial dilution to prepare a series of standard solutions with at least five different concentrations. Subsequently, specific primers designed for the target sequence are used to amplify each standard under identical quantitative PCR (qPCR) conditions. The cycle threshold (Ct) value, which corresponds to the cycle number at which the fluorescence signal reaches a predefined threshold, is recorded for each reaction. Finally, a standard curve is generated by performing linear regression analysis, plotting the logarithm of the initial standard concentration against the corresponding Ct value. This curve can then be applied for absolute quantification analysis of unknown samples in subsequent experiments.

Supplementary Figure S5

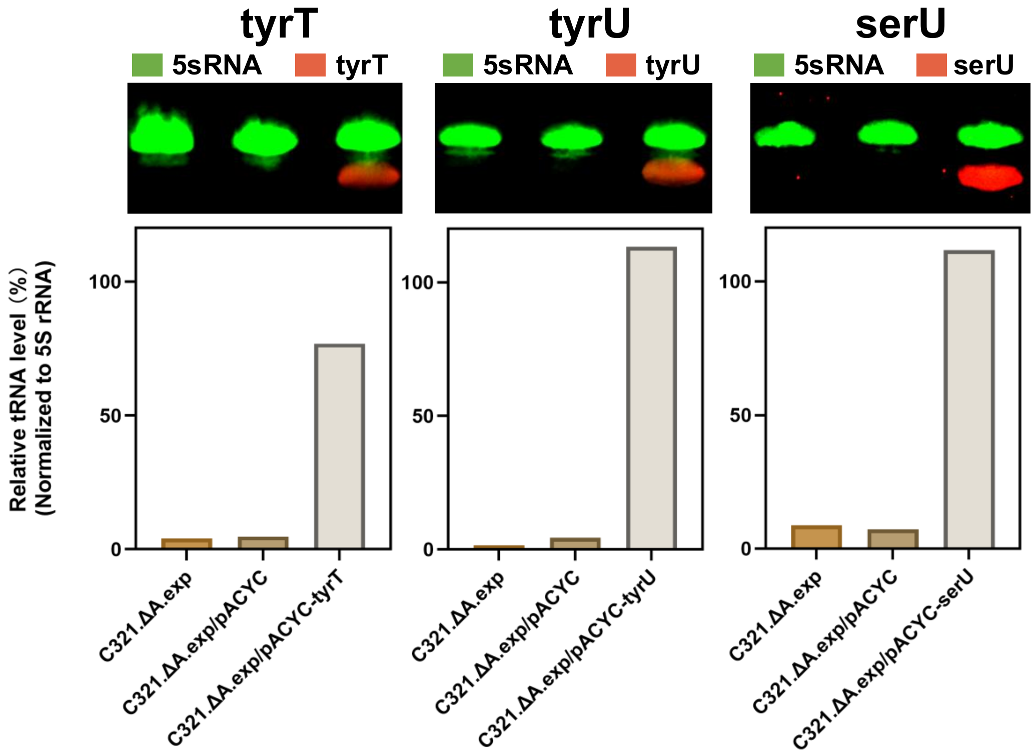

The expression levels of three sup-tRNAs(*tyrT/tyrU/serU*) were further validated using Northern blot.

Supplementary Figure S6

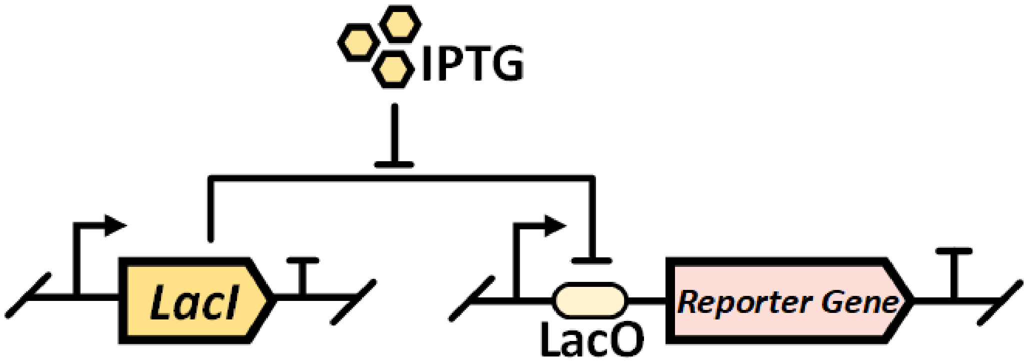

Schematic diagram of the regulation principle of biosensors based on LacI.
In the absence of an inducer (IPTG), the LacI protein binds to the operator sequence (*lacO*), thereby inhibiting transcription of the downstream reporter gene. When an inducer is present, it binds to LacI and induces a conformational change, causing LacI to dissociate from the DNA and enabling expression of the reporter gene. This mechanism achieves the sensing function of converting a chemical signal into a gene expression response.

Supplementary Figure S7

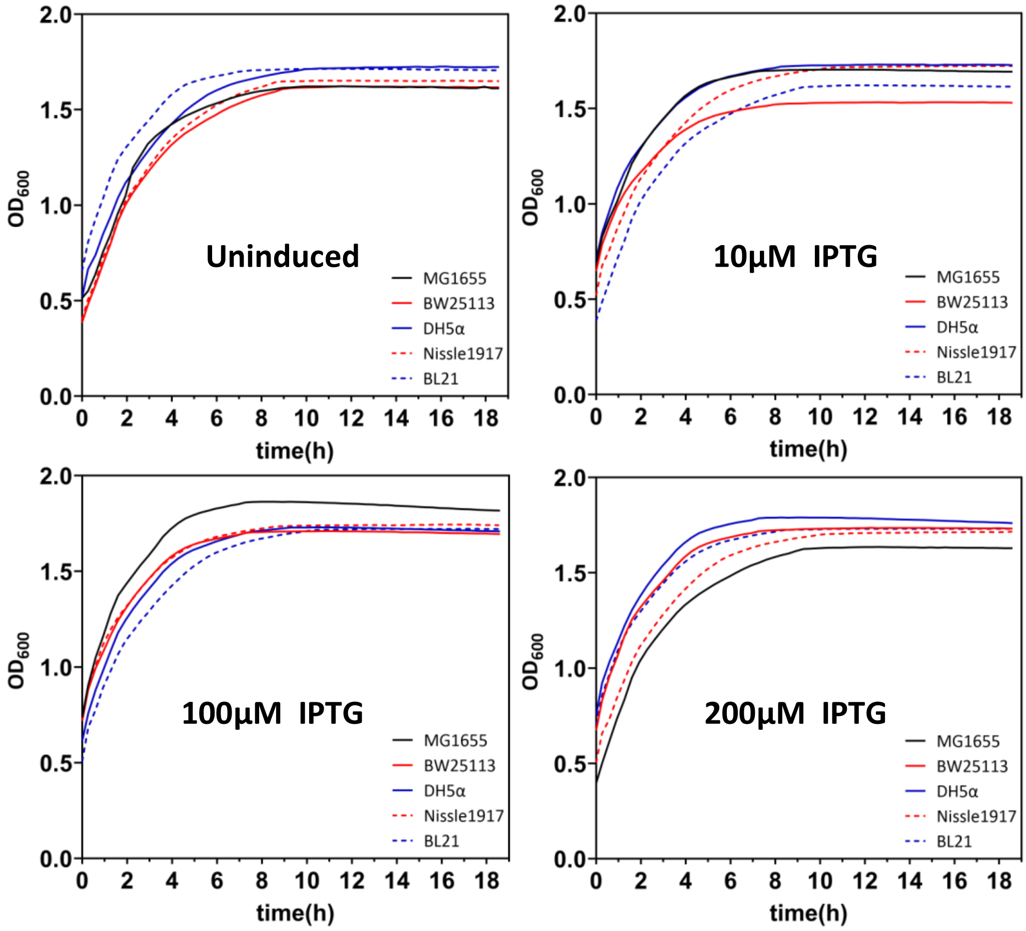

Growth profiles of strains MG1655, BW25113, DH5α, Nissle 1917, and BL21 in microplates under varying IPTG concentrations.

Supplementary Figure S8

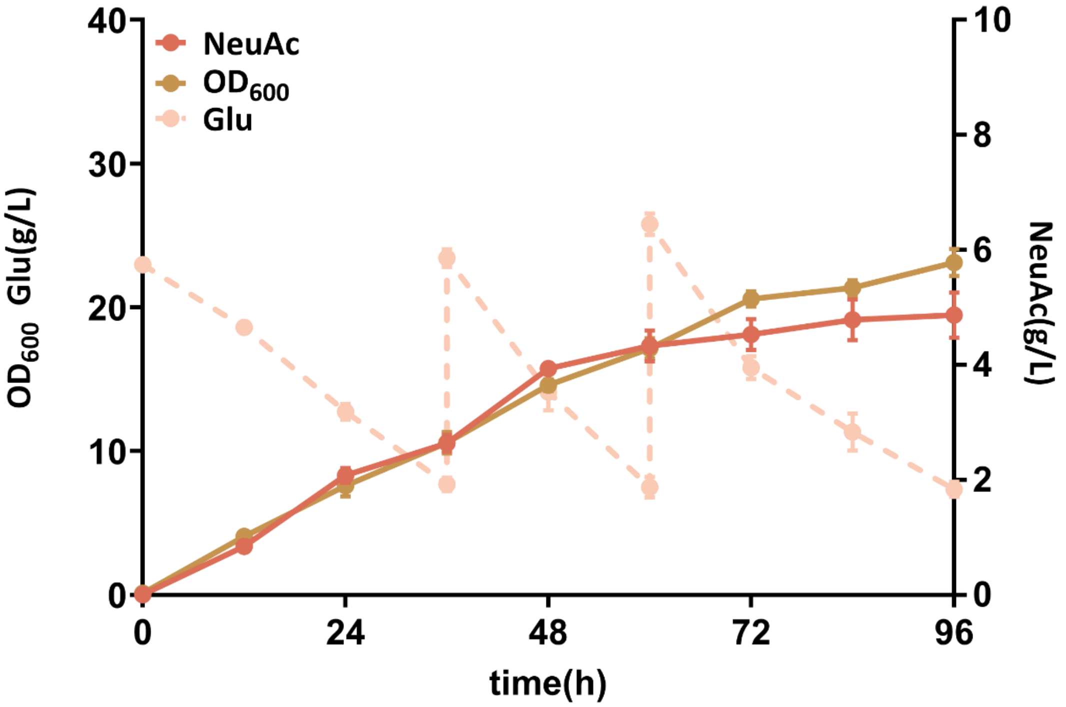

Fermentation profiles of strain DN5 (*ΔpykA ΔpykF*) in shake flasks over 96 h: NeuAc production, OD_{600}, and sugar consumption.

Supplementary Figure S9

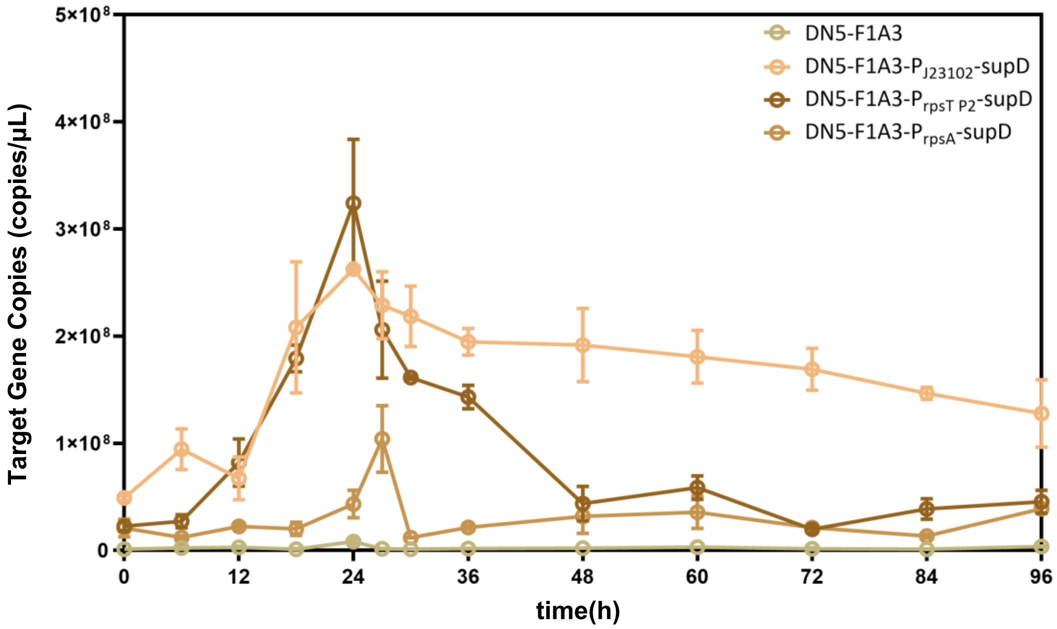

The abundance of supD under different promoters during shake flask fermentation. Absolute quantitative RT-PCR was performed to measure the supD transcript levels in DN5-F1A3, DN5-F1A3-P_{J23102}-*supD* (constitutive expression), DN5-F1A3-P_{rpsA}-*supD*, and DN5-F1A3-P_{rpsT} P2-*supD* strains during fermentation. The supD abundance under the control of GPPs (P_{rpsA} and $P_{rpsT\ P2}$) exhibited a clear rise-and-fall pattern along with cell growth, whereas no such trend was observed in the control strains (DN5-F1A3 and the constitutive expression strain).

Supplementary Figure S10

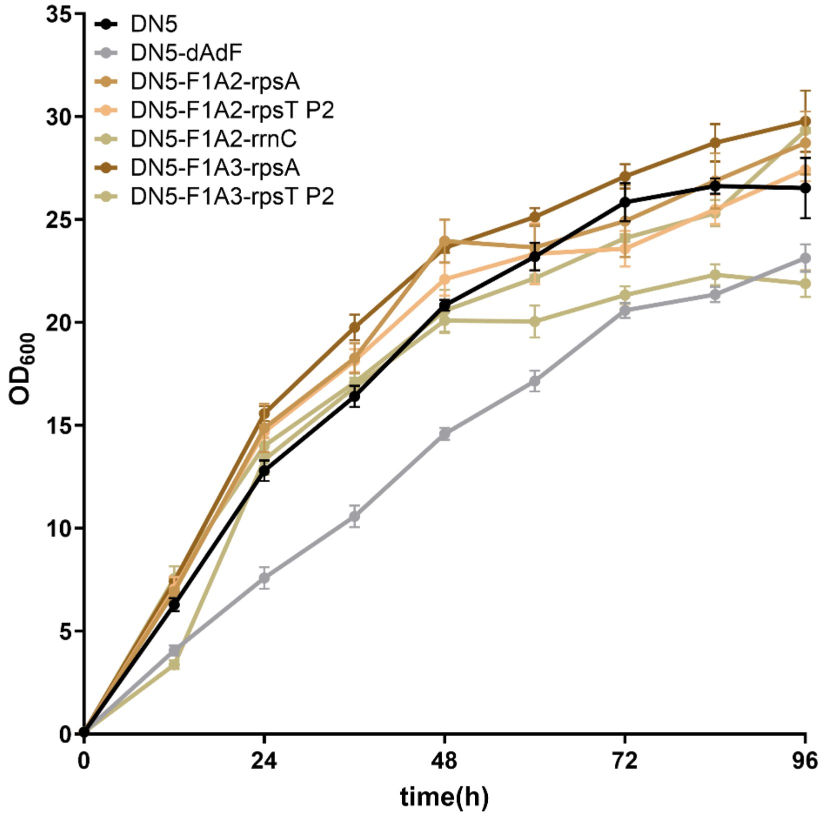

The growth of DN5 and its derivatives during the 96-hour fermentation process.

Supplementary Table S1. The strains used in this experiment

Strain	Description	Source
C321.ΔA.exp	E. coli MG1655 derivative, *Δ*(*ybhB-bioAB*)::*[lcI857 N(cro-ea59)*::*tetR-bla*] *ΔprfA ΔmutS*::*zeoR*; all 321 UAG codons changed to UAA	The C321.ΔA.exp strain was purchase from Addgene
MG1655	F^- λ^- $ilvG^-$ rfb-50 rph-1 Δ(*gpt-proA*)62	Lab storage
BW25113	F^- Δ(*araD-araB*)567 ΔlacZ4787(::*rrnB*-3) λ^- rph-1 Δ(*rhaD-rhaB*)568 hsdR514	Lab storage
DH5α	F^- φ80dlacZΔM15 Δ(*lacZYA-argF*)U169 *recA*1 *endA*1 *hsdR*17(rK^- mK^+) *supE*44 *thi*-1 *gyrA*96 *relA*1	Lab storage
Nissle1917	F^- Δ*thyA* Δ*endA* Δ*astA* $microcin^+$ sfa/foc^+ *hsdR*MS^- λ(DE3)	Lab storage
BL21	F^- *ompT gal dcm lon hsdSB*(rB^- mB^-) λ(DE3 [*lacI lacUV*5-T7 gene 1 ind1 sam7 nin5])	Lab storage
DN5	DH5α *Δnag ΔackA ΔpoxB ΔldhA Δnan*	Lab storage
DN5ΔpykAΔpykF	DH5α *Δnag ΔackA ΔpoxB ΔldhA ΔnanΔpykAΔpykF*	This study
DN5-F1	DN5 derivative, Using TAG instead of the second codon of *pykF*	This study
DN5-F2	DN5 derivative, Using TAG instead of the second and third codons of *pykF*	This study
DN5-F3	DN5 derivative, Using TAG instead of the second to fourth codons of *pykF*	This study
DN5-A1	DN5 derivative, Using TAG instead of the second codon of *pykA*	This study
DN5-A2	DN5 derivative, Using TAG instead of the second and third codons of *pykA*	This study
DN5-A3	DN5 derivative, Using TAG instead of the second to fourth codons of *pykA*	This study
DN5-F1A2	DN5 derivative, Using TAG instead of the second and third codons of *pykA* and the second codon of *pykF*	This study
DN5-F1A3	DN5 derivative, Using TAG instead of the second to fourth codons of *pykA,* and the second codon of *pykF*	This study

Supplementary Table S2. The Primers used in this experiment

Plasmid	Description	Source
pEcCas	Constitutive expression of Cas9 and inducible expression of λ-Red recombination systems	(1)
pEcgRNA-A	Derived from pEcgRNA, target *pykA* in E. coli DH5α	(1)
pEcgRNA-F	Derived from pEcgRNA, target *pykF* in E. coli DH5α	(1)
pBR322-sfGFP^{TAG}	Derived from pBR322, PJ23102-sfGFP^{TAG}-TrrnB	This study
P15A-Ala	Derived from pACYC, PJ23102-tRNA$^{Ala}_{CUA}$ -TrrnB	This study
P15A-Arg	Derived from pACYC, PJ23102-tRNA$^{Arg}_{CUA}$ -TrrnB	This study
P15A-Asn	Derived from pACYC, PJ23102-tRNA$^{Asn}_{CUA}$ -TrrnB	This study
P15A-Asp	Derived from pACYC, PJ23102-tRNA$^{Asp}_{CUA}$ -TrrnB	This study
P15A-Cys	Derived from pACYC, PJ23102-tRNA$^{Cys}_{CUA}$ -TrrnB	This study
P15A-Gln	Derived from pACYC, PJ23102-tRNA$^{Gln}_{CUA}$ -TrrnB	This study
P15A-Glu	Derived from pACYC, PJ23102-tRNA$^{Glu}_{CUA}$ -TrrnB	This study
P15A-Gly	Derived from pACYC, PJ23102-tRNA$^{Gly}_{CUA}$ -TrrnB	This study
P15A-His	Derived from pACYC, PJ23102-tRNA$^{His}_{CUA}$ -TrrnB	This study
P15A-Ile	Derived from pACYC, PJ23102-tRNA$^{Ile}_{CUA}$ -TrrnB	This study
P15A-Leu	Derived from pACYC, PJ23102-tRNA$^{Leu}_{CUA}$ -TrrnB	This study
P15A-Lys	Derived from pACYC, PJ23102-tRNA$^{Lys}_{CUA}$ -TrrnB	This study
P15A-Met	Derived from pACYC, PJ23102-tRNA$^{Met}_{CUA}$ -TrrnB	This study
P15A-Phe	Derived from pACYC, PJ23102-tRNA$^{Phe}_{CUA}$ -TrrnB	This study
P15A-Pro	Derived from pACYC, PJ23102-tRNA$^{Pro}_{CUA}$ -TrrnB	This study
P15A-Ser	Derived from pACYC, PJ23102-tRNA$^{Ser}_{CUA}$ -TrrnB	This study
P15A-Thr	Derived from pACYC, PJ23102-tRNA$^{Thr}_{CUA}$ -TrrnB	This study
P15A-Trp	Derived from pACYC, PJ23102-tRNA$^{Trp}_{CUA}$ -TrrnB	This study
P15A-Tyr	Derived from pACYC, PJ23102-tRNA$^{Tyr}_{CUA}$ -TrrnB	This study
P15A-Val	Derived from pACYC, PJ23102-tRNA$^{Val}_{CUA}$ -TrrnB	This study
P15A-Tyr(TAT)	Derived from pACYC, PJ23102-tRNA$^{Tyr}_{CUA}$ -TrrnB	This study
P15A-Leu(TTG)	Derived from pACYC, PJ23102-tRNA$^{Leu}_{CUA}$ -TrrnB	This study
P15A-Lys(AAG)	Derived from pACYC, PJ23102-tRNA$^{Lys}_{CUA}$ -TrrnB	This study
P15A-Glu(GAG)	Derived from pACYC, PJ23102-tRNA$^{Glu}_{CUA}$ -TrrnB	This study
pBR322-LacO-sfGFP^{TAG-1}	Derived from pBR322, Ptrc-LacO-sfGFP^{TAG}-TrrnB, replacing the second codon of sfGFP with TAG	This study
pBR322-LacO-sfGFP^{TAG-2}	Derived from pBR322, Ptrc-LacO-sfGFP^{TAG}-TrrnB, replacing the second and third codons of sfGFP with TAG	This study
pBR322-LacO-sfGFP^{TAG-3}	Derived from pBR322, Ptrc-LacO-sfGFP^{TAG}-TrrnB, replacing the second to fourth codon of sfGFP with TAG	This study
pBR322-LacO-sfGFP^{TAG-4}	Derived from pBR322, Ptrc-LacO-sfGFP^{TAG}-TrrnB, replacing the second to fifth codon of sfGFP with TAG	This study
pBR322-LacO-	Derived from pBR322, Ptrc-LacO-sfGFP^{TAG}-TrrnB,	This study

$sfGFP^{TAG-5}$	replacing the second to sixth codon of sfGFP with TAG	
pBR322-LacO-$sfGFP^{TAG-6}$	Derived from pBR322, Ptrc-LacO-$sfGFP^{TAG}$-TrrnB, replacing the second to seventh codon of sfGFP with TAG	This study
P15A-supD	Derived from pACYC, PJ23102-$tRNA^{Ser}_{CUA}$ -TrrnB	This study
P15A-supT	Derived from pACYC, PJ23102-$tRNA^{Tyr}_{CUA}$ (TAT)-TrrnB	This study
P15A-supC	Derived from pACYC, PJ23102-$tRNA^{Tyr}_{CUA}$ (TAC)-TrrnB	This study
P15A-2*supD	Derived from pACYC, PJ23102-2*$tRNA^{Ser}_{CUA}$ -TrrnB	This study
P15A-2*supT	Derived from pACYC, PJ23102-2*$tRNA^{Tyr}_{CUA}$ (TAT)-TrrnB	This study
P15A-2*supC	Derived from pACYC, PJ23102-2*$tRNA^{Tyr}_{CUA}$ (TAC)-TrrnB	This study
rpsL-sfGFP	Derived from pBR322, PrpsL -sfGFP-TrrnB	This study
rpsA-sfGFP	Derived from pBR322, PrpsA-sfGFP-TrrnB	This study
rpsT P1-sfGFP	Derived from pBR322, PrpsT P1-sfGFP-TrrnB	This study
rpsT P2-sfGFP	Derived from pBR322, PrpsT P2-sfGFP-TrrnB	This study
rrnC-sfGFP	Derived from pBR322, PrrnC-sfGFP-TrrnB	This study
rpsL-supD	Derived from pACYC, PrpsL -$tRNA^{Ser}_{CUA}$ -TrrnB	This study
rpsA-supD	Derived from pACYC, PrpsA-$tRNA^{Ser}_{CUA}$ -TrrnB	This study
rpsT P1-supD	Derived from pACYC, PrpsT P1 -$tRNA^{Ser}_{CUA}$ -TrrnB	This study
rpsT P2-supD	Derived from pACYC, PrpsT P2 -$tRNA^{Ser}_{CUA}$ -TrrnB	This study
rrnC-supD	Derived from pACYC, PrrnC -$tRNA^{Ser}_{CUA}$ -TrrnB	This study
pBac43	pTrc99a, harboring *neuB, age, GNA*1 and mutant *glmS* gene	This study

Supplementary Table S3. Optimal codons in *Escherichia coli*

Amino acid	Optimal Codons
Ala(A)	GCG
Arg(R)	CGC
Asn(N)	AAC
Asp(D)	GAC
Cys(C)	UGC
Gln(Q)	CAG
Glu(E)	GAA
Gly(G)	GGC
His(H)	CAC
Ile(I)	AUU
Leu(L)	CUG
Lys(K)	AAA
Met(M)	AUG
Phe(P)	UUC
Pro(P)	CCG
Ser(S)	UCG
Thr(T)	ACC
Trp(W)	UGG
Tyr(Y)	UAC
Val(V)	GUG

Supplementary Table S4. List of tRNA genes engineered for amber suppression in *E. coli.*

Amino Acid	Anticodon (Native)	Genomic Designation (from tRNAdb)	tRNA Gene Name	Rationale for Selection
**Ala**	TGC*3	tRNA-Ala-TGC-1-1	***alaT***	Decodes the optimal alanine codon in *E. coli*. (2)
	GGC*2	tRNA-Ala-GGC-1-1	*alaX*	
**Arg**	ACG*4	tRNA-Arg-ACG-1-1	***argQ***	Decodes the optimal arginine codons and is a major $tRNA^{Arg}$ in *E. coli*.
	CCG	tRNA-Arg-CCG-1-1	*argX*	
	CCT	tRNA-Arg-CCT-1-1	*argW*	
	TCT	tRNA-Arg-TCT-1-1	*argU*	
**Asn**	GTT*4	tRNA-Asn-GTT-1-1	***asnT***	Decodes the optimal asparagine codon and is one of the four identical $tRNA^{Asn}$ genes in *E. coli*. (3)
**Asp**	GTC*3	tRNA-Asp-GTC-1-1	***aspT***	Decodes the optimal aspartate codon and is one of the three identical $tRNA^{Asp}$ genes in *E. coli*.(4)
**Cys**	GCA	tRNA-Cys-GCA-1-1	***cysT***	Decodes the optimal cysteine codon and is the sole $tRNA^{Cys}$ gene in *E. coli*.(5)
**Glu**	TTC*4	tRNA-Glu-TTC-1-1	***gluU***	Decodes the optimal glutamate codon and is one of the four identical $tRNA^{Glu}$ genes in *E. coli*.(6)
**Gln**	CTG*2	tRNA-Gln-CTG-1-1	***glnX***	Decodes the optimal glutamine codon in *E. coli*. (7)
	TTG*2	tRNA-Gln-TTG-1-1	*glnU*	
**Gly**	GCC*4	tRNA-Gly-GCC-1-1	***glyV***	Decodes optimal glycine codons in *E. coli*.
	CCC	tRNA-Gly-CCC-1-1	*glyU*	
	TCC	tRNA-Gly-TCC-1-1	*glyT*	
**His**	GTG	tRNA-His-GTG-1-1	***hisR***	Decodes the optimal histidine codon and is the sole $tRNA^{His}$ gene in *E. coli*. (8)
**Ile**	GAT*3	tRNA-Ile-GAT-1-1	***ileU***	Decodes the optimal/high-frequency isoleucine codons and is the primary $tRNA^{Ile}$ in *E. coli*. (9)
	CAT	tRNA-Ile2-CAT-1-1	*ileX*	
	CAT	tRNA-Ile2-CAT-2-1	*ileY*	
**Leu**	CAG	tRNA-Leu-CAG-2-1	***leuP***	Decodes the optimal leucine codon in *E. coli*.
	CAG*3	tRNA-Leu-CAG-1-1	*leuQ*	
	GAG	tRNA-Leu-GAG-1-1	*leuU*	

	CAA	tRNA-Leu-CAA-1-1	*leuX*	
	TAA	tRNA-Leu-TAA-1-1	*leuZ*	
	TAG	tRNA-Leu-TAG-1-1	*leuW*	
**Lys**	TTT*6	tRNA-Lys-TTT-1-1	***lysQ***	Decodes the optimal lysine codon and is one of the six identical $tRNA^{Lys}$ genes in *E. coli*.(10)
**Met**	CAT*2	tRNA-Met-CAT-1-1	***metU***	Decodes the methionine codon and is one of the two identical $tRNA^{Met}$ genes in *E. coli*.
**Phe**	GAA*2	tRNA-Phe-GAA-1-1	***pheU***	Decodes the optimal phenylalanine codon and is one of the two identical $tRNA^{Phe}$ genes in *E. coli*.
**Pro**	CGG	tRNA-Pro-CGG-1-1	***proK***	Decodes the optimal proline codon in *E. coli*. (11)
	GGG	tRNA-Pro-GGG-1-1	*proL*	
	TGG	tRNA-Pro-TGG-1-1	*proM*	
**Ser**	CGA	tRNA-Ser-CGA-1-1	***serU***	Decodes optimal serine codons in *E. coli*.
	GGA*2	tRNA-Ser-GGA-1-1	*serX*	
	TGA	tRNA-Ser-TGA-1-1	*serT*	
	GCT	tRNA-Ser-GCT-1-1	*serV*	
**Thr**	GGT	tRNA-Thr-GGT-1-1	***thrT***	Decodes the optimal threonine codon in *E. coli*.
	GGT	tRNA-Thr-GGT-2-1	*thrV*	
	CGT	tRNA-Thr-CGT-1-1	*thrW*	
	TGT	tRNA-Thr-TGT-1-1	*thrU*	
**Trp**	CCA	tRNA-Trp-CCA-1-1	***trpT***	Decodes the optimal tryptophan codon and is the sole $tRNA^{Trp}$ gene in *E. coli*.
**Tyr**	GTA*2	tRNA-Tyr-GTA-2-1	***tyrT***	Decodes the optimal tyrosine codon in *E. coli*.
	GTA	tRNA-Tyr-GTA-1-1	*tyrU*	
**Val**	GAC	tRNA-Val-GAC-1-1	***valW***	Decodes a high-frequency valine codon and is one of the two $tRNA^{Val}$ isoacceptors in *E. coli* (selected for stable plasmid expression). (12)
	GAC	tRNA-Val-GAC-2-1	*valV*	
	TAC*5	tRNA-Val-TAC-1-1	*valX*	

Supplementary Table S5. Binding free energies between AARS and tRNA evaluated with MM/GBSA approach.

AARS	tRNA	Energy components[a]				
		ΔE_{ele}	ΔE_{vdW}	ΔG_{GB}	ΔG_{SA}	ΔG_{bind}[b]
AlaRS	WT	3710.61 ± 53.97	–230.23 ± 8.93	–3713.64 ± 5185	–31.61 ± 0.63	–264.88 ± 9.67
	Mod	3848.46 ± 74.58	–225.17 ± 9.32	–3838.51 ± 70.57	–31.23 ± 0.97	–246.45 ± 8.78
ArgRS	WT	1924.29 ± 47.43	–247.48 ± 9.50	–1906.43 ± 46.16	–35.97 ± 1.22	–265.59 ± 9.39
	Mod	1931.01 ± 45.27	–219.09 ± 10.76	–1907.21 ± 43.33	–31.87 ± 1.57	–227.16 ± 11.30
AsnRS	WT	3234.97 ± 46.34	–168.00 ± 7.21	–3222.42 ± 44.73	–25.71 ± 0.59	–181.16 ± 6.59
	Mod	3210.11 ± 37.76	–191.51 ± 7.66	–3202.84 ± 36.98	–27.04 ± 0.44	–211.28 ± 7.08
AspRS	WT	2180.90 ± 55.22	–235.48 ± 8.75	–2172.13 ± 53.82	–32.96 ± 0.64	–259.66 ± 8.03
	Mod	2197.51 ± 76.55	–184.47 ± 9.94	–2186.68 ± 74.40	–27.75 ± 1.00	–201.39 ± 9.49
CysRS	WT	2086.85 ± 48.35	–327.10 ± 9.43	–2076.50 ± 46.44	–44.48 ± 0.71	–361.23 ± 8.81
	Mod	2148.10 ± 34.71	–247.71 ± 8.08	–2131.08 ± 33.25	–36.34 ± 0.74	–267.02 ± 7.88
GlnRS	WT	581.73 ± 54.45	–318.50 ± 9.59	–573.63 ± 52.66	–44.15 ± 0.83	–354.55 ± 9.11
	Mod	636.26 ± 56.34	–320.69 ± 8.71	–631.02 ± 54.43	–43.89 ± 1.07	–359.35 ± 9.02
GluRS	WT	1636.11 ± 45.76	–277.05 ± 8.31	–1619.73 ± 44.76	–37.86 ± 0.91	–298.53 ± 8.02
	Mod	1589.38 ± 39.73	–253.95 ± 10.55	–1572.53 ± 38.46	–34.57 ± 0.90	–271.66 ± 10.63
GlyRS	WT	1741.58 ± 51.46	–153.68 ± 7.57	–1739.38 ± 49.27	–21.29 ± 0.92	–172.77 ± 7.75
	Mod	1640.53 ± 49.67	–141.02 ± 7.03	–1642.70 ± 48.24	–18.58 ± 0.87	–161.77 ± 6.92
HisRS	WT	–307.32 ± 28.62	–151.37 ± 10.41	287.47 ± 26.06	–25.65 ± 0.93	–196.88 ± 9.84
	Mod	–327.23 ± 29.47	–155.17 ± 7.52	316.63 ± 27.12	–25.11 ± 0.88	–190.88 ± 7.37
IleRS	WT	2266.93 ± 53.12	–283.22 ± 9.17	–2235.65 ± 51.23	–41.10 ± 1.06	–293.04 ± 9.72
	Mod	2283.97 ± 61.10	–247.31 ± 9.27	–2258.78 ± 58.79	–36.10 ± 0.92	–258.23 ± 9.72
LeuRS	WT	2492.13 ± 68.25	–285.51 ± 8.40	–2481.52 ± 65.84	–42.45 ± 1.00	–317.34 ± 8.39
(leuX)	Mod	2544.28 ± 60.22	–287.21 ± 12.07	–2531.42 ± 58.76	–43.08 ± 1.47	–317.43 ± 12.31
LeuRS	WT	2783.44 ± 82.83	–322.79 ± 8.67	–2763.38 ± 80.02	–48.00 ± 1.07	–350.74 ± 9.56
(leuP)	Mod	2630.44 ± 80.37	–326.52 ± 8.36	–2611.71 ± 87.79	–47.53 ± 1.42	–355.33 ± 10.34
LysRS	WT	5034.12 ± 69.93	–308.36 ± 9.62	–4989.73 ± 66.99	–42.28 ± 0.81	–306.25 ± 11.05
	Mod	4813.59 ± 62.79	–258.22 ± 8.35	–4791.24 ± 61.44	–36.18 ± 1.05	–272.04 ± 8.09
MetRS	WT	1358.72 ± 53.38	–288.05 ± 10.51	–1338.16 ± 51.65	–43.17 ± 1.52	–310.66 ± 11.01
	Mod	1556.18 ± 49.90	–287.79 ± 12.24	–1535.36 ± 48.89	–40.78 ± 1.24	–307.74 ± 11.72
PheRS	WT	6424.45 ± 72.72	–159.75 ± 12.63	–6403.80 ± 70.33	–23.24 ± 1.75	–162.34 ± 7.23
	Mod	6719.73 ± 64.79	–214.57 ± 11.39	–6686.09 ± 62.32	–31.07 ± 1.60	–212.00 ± 12.48
ProRS	WT	3952.75 ± 54.78	–177.50 ± 8.90	–3948.22 ± 52.90	–27.52 ± 0.97	–200.50 ± 9.04
	Mod	4145.17 ± 65.45	–154.24 ± 9.36	–4121.37 ± 62.69	–23.96 ± 2.64	–154.40 ± 9.21
SerRS	WT	3236.16 ± 56.88	–132.67 ± 8.38	–3223.60 ± 55.32	–18.74 ± 1.40	–138.84 ± 7.13
	Mod	3258.08 ± 59.21	–177.56 ± 9.75	–3253.45 ± 58.02	–25.72 ± 0.94	–198.65 ± 9.59

ThrRS	WT	3050.71 ± 56.51	–295.60 ± 9.57	–3039.33 ± 55.44	–42.56 ± 0.77	–326.78 ± 8.81
	Mod	3101.73 ± 49.47	–228.41 ± 8.59	–3080.88 ± 48.41	–33.75 ± 0.99	–241.31 ± 8.56
TrpRS	WT	275.70 ± 23.93	–65.86 ± 4.89	–280.65 ± 22.28	–9.53 ± 0.70	–80.34 ± 4.51
	Mod	293.57 ± 34.11	–83.48 ± 6.85	–294.44 ± 32.24	–11.99 ± 1.36	–96.34 ± 8.62
TyrRS	WT	1210.90 ± 57.56	–107.88 ± 7.88	–1197.90 ± 56.42	–17.64 ± 1.47	–112.53 ± 8.63
(tyrT)	Mod	960.85 ± 49.18	–101.68 ± 7.32	–957.78 ± 46.14	–19.02 ± 1.10	–117.64 ± 9.12
TyrRS	WT	1160.22 ± 52.13	–123.79 ± 9.89	–1151.09 ± 49.32	–19.11 ± 1.64	–133.78 ± 9.16
(tyrU)	Mod	818.93 ± 52.06	–169.05 ± 10.26	–818.78 ± 59.43	–26.78 ± 1.39	–195.68 ± 8.92
ValRS	WT	3200.31 ± 61.28	–275.82 ± 9.18	–3177.49 ± 58.87	–39.66 ± 1.16	–292.66 ± 8.90
	Mod	3260.32 ± 70.53	–209.04 ± 8.85	–3235.440 ± 68.63	–30.47 ± 1.13	–214.60 ± 8.52

[a] Energies are in kcal·mol^{-1}.

[b] $\Delta G_{bind} = \Delta E_{ele} + \Delta E_{vdW} + \Delta G_{GB} + \Delta G_{SA}$.

Supplementary Table S6. Binding free energies calculated with the MM/GBAS approach.

tRNA	Anticodon-dependent or independent recognition	aaRS Class	tRNA-aaRS-original	tRNA-aaRS-modified	$\Delta\Delta G_{bind}$
tyrU	Dependent	Class I	-133.78 ± 9.16	-195.68 ± 8.92	61.9
serU	Independent	Class II	-138.84 ± 7.13	-198.65 ± 9.59	59.81
pheU	Dependent	Class II	-162.34 ± 7.23	-212.00 ± 12.48	49.66
trpT	Dependent	Class I	-80.34 ± 4.51	-96.34 ± 8.62	16.00
asnT	Dependent	Class II	-181.16 ± 6.59	-211.28 ± 7.08	30.12
tyrT	Dependent	Class I	-112.53 ± 8.63	-117.64 ± 9.12	5.11
glnX	Dependent	Class I	-354.55 ± 9.11	-359.35 ± 9.02	4.80
leuP	Independent	Class I	-350.74 ± 9.56	-355.33 ± 10.34	4.59
leuX	Independent	Class I	-317.34 ± 8.39	-317.43 ± 12.31	0.09
metU	Dependent	Class I	-310.66 ± 11.01	-307.74 ± 11.72	-2.92
hisR	Dependent	Class II	-196.88 ± 9.84	-190.88 ± 7.37	-6.00
glyV	Dependent	Class II	-172.77 ± 7.75	-161.77 ± 6.92	-11.00
alaT	Independent	Class II	-264.88 ± 9.67	-246.45 ± 8.78	-18.43
gluU	Dependent	Class I	-298.53 ± 8.02	-271.66 ± 10.63	-26.87
lysQ	Dependent	Class II	-306.25 ± 11.05	-272.04 ± 8.09	-34.21
ileX	Dependent	Class I	-293.04 ± 9.72	-258.23 ± 9.72	-34.81
argQ	Dependent	Class I	-265.59 ± 9.39	-227.16 ± 11.30	-38.43
aspT	Dependent	Class II	-259.66 ± 8.03	-201.39 ± 9.49	-58.27
proK	Dependent	Class II	-200.50 ± 9.04	-154.40 ± 9.21	-46.1
cysT	Dependent	Class I	-361.23 ± 8.81	-267.02 ± 7.88	-94.21
thrT	Dependent	Class II	-326.78 ± 8.81	-241.31 ± 8.56	-85.47
valW	Dependent	Class I	-292.66 ± 8.90	-214.60 ± 8.52	-78.06

The change in binding affinity due to the mutation was defined as $\Delta\Delta G_{bind}=\Delta G_{bind}$(tRNA-original)-$\Delta G_{bind}$(tRNA-modified), where positive values indicate enhanced binding of the sup-tRNA.

Supplementary Table S7. Fermentation parameters of NeuAc production in *E. coli* strains under different sup-tRNA regulatory strategies

Strain	Regulation	Titer (g/L)	Overall Yield (g/g glucose)	Specific Yield (mg/g glucose/g CDW)	Yield improvement vs DN5 (%)
DN5	_	5.33	0.0851	8.04	_
DN5-dAdF	_	4.86	0.0905	9.80	-8.81%
DN5-F1A3	P_{rpsA}-supD	6.72	0.101	8.29	19%
DN5-F1A3	$P_{rpsT-P2}$-supD	8.82	0.141	12.1	66%

References

1. Li, Q., Sun, B., Chen, J., Zhang, Y., Jiang, Y. and Yang, S. (2021) A modified pCas/pTargetF system for CRISPR-Cas9-assisted genome editing in Escherichia coli. *Acta Biochim Biophys Sin (Shanghai)*, **53**, 620-627.
2. Ma, C., Kudlicki, W., Odom, O.W., Kramer, G. and Hardesty, B. (1993) In vitro protein engineering using synthetic tRNA(Ala) with different anticodons. *Biochemistry*, **32**, 7939-7945.
3. Li, S., Pelka, H. and Schulman, L.H. (1993) The anticodon and discriminator base are important for aminoacylation of Escherichia coli tRNA(Asn). *J Biol Chem*, **268**, 18335-18339.
4. Martin, F., Barends, S. and Eriani, G. (2004) Single amino acid changes in AspRS reveal alternative routes for expanding its tRNA repertoire in vivo. *Nucleic Acids Res*, **32**, 4081-4089.
5. Komatsoulis, G.A. and Abelson, J. (1993) Recognition of tRNA(Cys) by Escherichia coli cysteinyl-tRNA synthetase. *Biochemistry*, **32**, 7435-7444.
6. Sekine, S., Nureki, O., Shimada, A., Vassylyev, D.G. and Yokoyama, S. (2001) Structural basis for anticodon recognition by discriminating glutamyl-tRNA synthetase. *Nat Struct Biol*, **8**, 203-206.
7. Ibba, M., Hong, K.W., Sherman, J.M., Sever, S. and Söll, D. (1996) Interactions between tRNA identity nucleotides and their recognition sites in glutaminyl-tRNA synthetase determine the cognate amino acid affinity of the enzyme. *Proc Natl Acad Sci U S A*, **93**, 6953-6958.
8. Englert, M., Vargas-Rodriguez, O., Reynolds, N.M., Wang, Y.S., Söll, D. and Umehara, T. (2017) A genomically modified Escherichia coli strain carrying an orthogonal E. coli histidyl-tRNA synthetase•tRNA(His) pair. *Biochim Biophys Acta Gen Subj*, **1861**, 3009-3015.
9. Muramatsu, T., Nishikawa, K., Nemoto, F., Kuchino, Y., Nishimura, S., Miyazawa, T. and Yokoyama, S. (1988) Codon and amino-acid specificities of a transfer RNA are both converted by a single post-transcriptional modification. *Nature*, **336**, 179-181.
10. Tamura, K., Himeno, H., Asahara, H., Hasegawa, T. and Shimizu, M. (1992) In vitro study of E.coli tRNA(Arg) and tRNA(Lys) identity elements. *Nucleic Acids Res*, **20**, 2335-2339.
11. Liu, H., Peterson, R., Kessler, J. and Musier-Forsyth, K. (1995) Molecular recognition of tRNA(Pro) by Escherichia coli proline tRNA synthetase in vitro. *Nucleic Acids Res*, **23**, 165-169.
12. Horowitz, J., Chu, W.C., Derrick, W.B., Liu, J.C., Liu, M. and Yue, D. (1999) Synthetase recognition determinants of E. coli valine transfer RNA. *Biochemistry*, **38**, 7737-7746.
